# The effects of orthobiologics in the treatment of tendon pathologies: a systematic review of preclinical evidence

**DOI:** 10.1186/s40634-022-00468-w

**Published:** 2022-04-08

**Authors:** Marco Viganò, Enrico Ragni, Antonio Marmotti, Laura de Girolamo

**Affiliations:** 1 Orthopaedics biotechnology Lab, IRCCS Istituto Ortopedico Galeazzi, Via Riccardo Galeazzi 4, 20161 Milan, Italy; 2 San Luigi Gonzaga Hospital, Orthopedics and Traumatology Department, University of Turin - Medical School, Turin, Italy

**Keywords:** PRP, Bone marrow, Adipose tissue, Stromal vascular fraction, Tendon, Tendinopathy, Rotator cuff, Orthobiologics, Regenerative medicine, Animal models

## Abstract

**Purpose:**

The aim of this systematic review is to explore the current available knowledge about tendon disorders and orthobiologics derived by preclinical experiments to evaluate their role and efficacy in the different stages and conditions related to the tendon healing processes.

**Methods:**

The systematic review was performed according to the PRISMA guidelines. Different electronic databases (MEDLINE, Web of Science, EMBASE) were searched for studies investigating orthobiologics (PRP and cell-based products from adipose tissue or bone marrow) in animal models or veterinary clinical trials for tendon pathologies (complete/partial tendon ruptures, rotator cuff tears, tendinopathy, enthesis-related injuries). Data regarding the specific product used, the treatment site/pathology, the host and the model were collected. The results were classified into the following categories: histological, biomechanical, molecular and imaging.

**Results:**

A large pool of preclinical studies on tendon disorders have been found on platelet-rich plasma (PRP), while data about stromal vascular fraction (SVF) and bone marrow concentrate (BMAC) are still limited and frequently focused on expanded cells, rather than orthobiologics prepared at the point of care.

The effect of PRP is related to an acceleration of the healing process, without improvements in the final structure and properties of repaired tendon. Cell-based products have been reported to produce more durable results, but the level of evidence is currently insufficient to draw clear indications.

**Conclusions:**

The preclinical results about orthobiologics applications to tendon pathologies would support the rationale of their clinical use and encourage the performance of clinical trials aimed to confirm these data in human subjects.

**Supplementary Information:**

The online version contains supplementary material available at 10.1186/s40634-022-00468-w.

## Background

Tendon injuries, especially those affecting Achilles, forearm extensor, patellar and rotator cuff tendons, represent a very common condition [9, 55, 112]. At present, management strategies are limited in terms of both success and scientific robustness [3]. The term “tendon disorders” includes a wide range of pathologies, including partial or complete ruptures (deriving from traumatic injuries or late-stage degenerative conditions), inflammation with early stage tissue degeneration (often associated with overuse) [2, 67], and, thus, the treatment choice may significantly vary depending on the specific condition. For example, ruptures require in most cases a surgical intervention, while other conditions may benefit from conservative therapies in order to control symptoms and possibly halt the degenerative process [28, 58]. In the scenario of non-surgical approaches, regenerative medicine aims to promote and sustain tissue repair by exploitation of the self-healing ability of the body [6, 25]. Regenerative medicine comprises a number of approaches, including the application of orthobiologics, i.e. blood-derived and cell cell-based products (from bone marrow and adipose tissue-derived), that represent a ready-to-use and cutting-edge strategy to enhance tissue healing [1, 23]. Blood-derived products comprise plasma- or serum-based whole blood derivatives. In general, they are well tolerated and provide reduction of pain and inflammation. The mechanism of action of these products relies on their content of cytokines and growth factors that stimulates cell proliferation and modulates the inflammatory response [34]. During the last 30 years, several formulations have been developed, all with different properties. Autologous PRP is the most used, but even considering only this product, preparation protocols may significantly vary, depending on the depletion or concentration of leukocytes and the activation of platelets [5]. Among cell-based products, bone marrow represents the most traditional source of mesenchymal stem/stromal cells (or Medicinal Signaling Cells, MSCs) in the adult human body [39]. The rationale of MSCs use in regenerative medicine relies on their ability to contribute to tissue healing through restoration of tissue homeostasis [16, 84]. In fact, the MSCs are able to release paracrine effectors promoting healing by the modulation of the response to injury of tissue resident and immune cells response [113]. These properties are shared by all the MSCs, regardless of their origin [4, 98, 106]. Adipose tissue popularity as a source for regenerative medicine products is gaining momentum thanks to its relative abundance and ease of harvest. Bone marrow aspirate concentrate (BMAC), freshly isolated stromal vascular fraction (SVF), as well as micro/nano-fragmented adipose tissue, represent intraoperative and minimally manipulated solutions for the application of MSCs-based therapies. Compared to culture-expanded cells, characterized by a higher homogeneity, these products includes different cell types together with MSCs, such as hematopoietic cells from bone marrow, or epithelial cells and pre-adipocytes from adipose tissue [12]. Other approaches, such tissue engineering techniques [91] and products derived from embryonic annexes [44, 121] have been proposed, but their use in these conditions is less represented in current literature and the analysis of these techniques is beyond the aims and scope of the present document. 

While orthobiologics have been used extensively for the treatment of bone and cartilage diseases, the application to tendon disorders of this large variety of products is under-investigated. The aim of this systematic review is to explore the current available knowledge about orthobiologics for the treatment of tendon disorders in in vivo preclinical studies to evaluate their efficacy in the different tendon conditions. Given the little knowledge available from the clinical trials in this field, the evidence provided by preclinical studies may allow for the identification of the most promising applications in each different condition, in order to guide further clinical research on a subset of specific products and strategies.

## Methods

### Sources and study selection

**Fig. 1:**
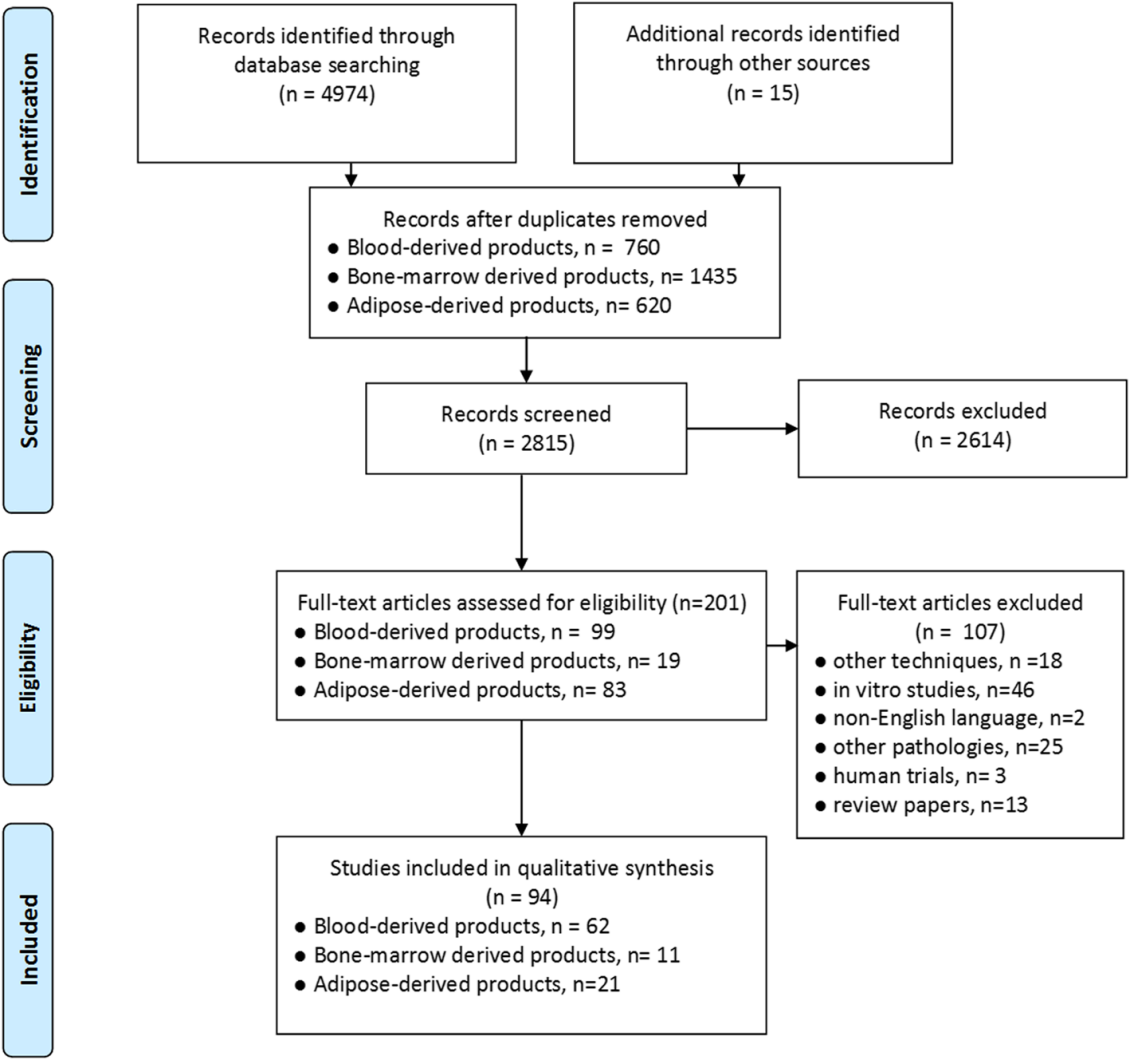
PRISMA flow-chart summarizing study selection process

This systematic review was prepared according to the PRISMA guidelines and Cochrane Collaboration methodology [88]. Three electronic databases (MEDLINE, Web of Science, EMBASE) were searched on September 30th, 2021. In addition, further reports were manually identified in the reference list of previously published systematic reviews addressing this topic. Figure 1 reports the flowchart representing the studies selection process. Supplementary Table 1 provides the list of keywords and MeSH terms used for data retrieval. 

The screening process was focused on identifying preclinical studies or veterinary clinical trials investigating the effects of blood-derived products, cell-based products derived from adipose tissue and bone marrow in tendon disorders. The following reports were excluded during the screening process: in vitro studies or clinical trials on human subjects; studies evaluating the effects of orthobiologics on other tissues; studies investigating other techniques; documents written in languages other than English. Study selection was performed by two independent researchers. Figure 1 reports the PRISMA flow chart of the study selection process.

### Blood-derived products

The search for articles reporting preclinical studies concerning blood-derived products and tendons retrieved 89 papers after title/abstract screening. Further 10 papers were identified by manual search within the references of full-texts included in the analysis. They were all evaluated by abstract screening, and 37 were excluded because considered non-inherent for various reasons or because they did not satisfy inclusion/exclusion criteria. Then, 62 studies were included in the qualitative analysis.

### Bone marrow-derived products

The search for studies reporting the efficacy of cultured mesenchymal stem cells or bone marrow aspirate concentrates (BMAC) in tendon conditions retrieved 19 documents. Of these, 8 were excluded after title/abstract screening, resulting in 11 studies available for analysis.

### Adipose tissue-derived products

Seventy-eight studies were identified during database search after title/abstract screening and 5 more were found among references of full-texts included in the analysis. Sixty-two studies were excluded because considered non-inherent for various reasons or because they did not satisfy inclusion/exclusion criteria, resulting in 21 studies included in the analysis.

### Data collection and risk of bias evaluation

Product specifications, treatment site (or specific pathology), type of host and model, and experimental results (histological, biomechanical, molecular and imaging findings) were collected for all studies included in the qualitative analysis.

Risk of bias was determined for all studies using SYRCLE’s tool for animal studies. Two independent investigators rated the risk of bias for each study as low, high or unclear, depending on specific items dedicated to the identification of selection, performance, detection, attrition and reporting biases [52].

## Results

### Blood-derived products

#### Selected studies and overall evaluation

**Fig. 2:**
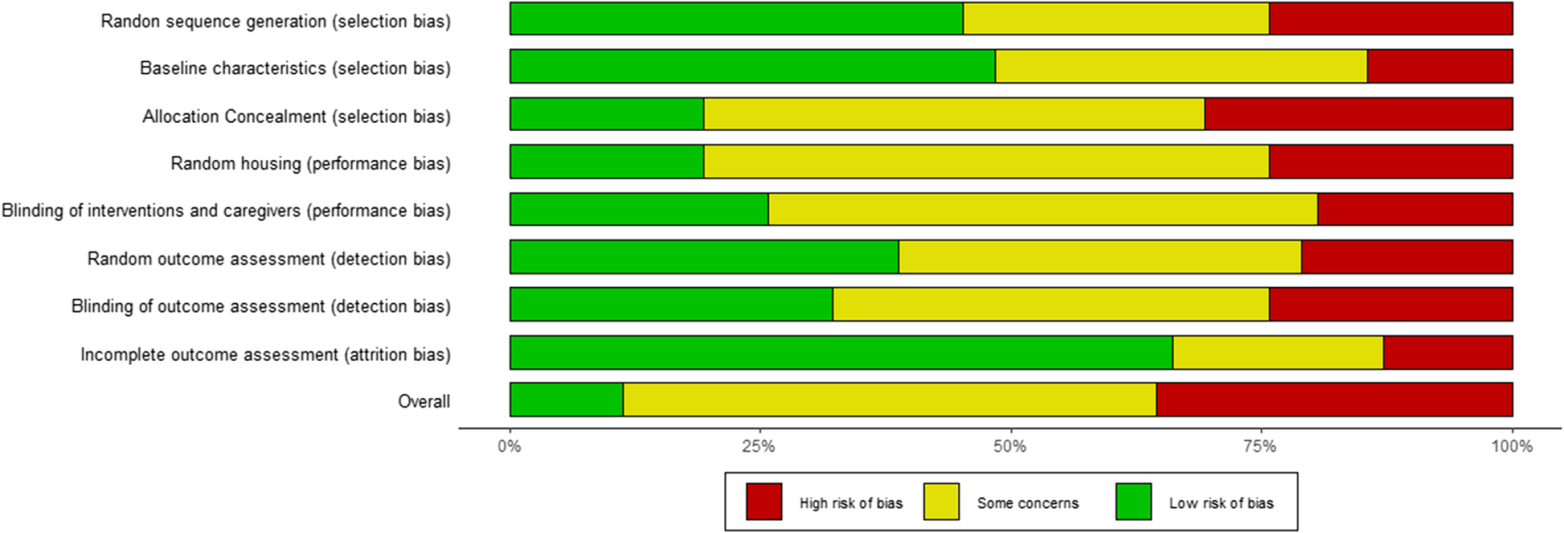
SYRCLE’s risk of bias assessment for in vivo studies investigating the use of blood-derived products for the treatment of tendon pathologies

Among the 62 selected studies investigating the effects of blood-derived products, the vast majority were based on rat (*n* = 28) or rabbit (*n* = 26) models. Three and 2 studies respectively used horse and sheep models, while only one study was conducted in mice. In addition, two veterinary trials involving dogs were selected. Thirty-three studies used models of tendon ruptures, 8 addressed spontaneous or experimentally-induced tendinopathy, 10 investigated the rotator cuff tears and 6 studies involved tendon-bone junctions. The effect of PRP alone was evaluated in 57 studies, while 9 reports on PRP in addition to other therapies (drugs or cells). In 6 cases, PRP was used in combination with scaffolds. Platelet-rich fibrin (PRF) and plasma rich in growth factors (PRGF) were evaluated in 4 and 1 studies, respectively. According to SYRCLE’s tool, 22 studies were at high risk of bias, 9 due to the lack of control group and 13 due to lack of randomization and/or blinding of caregivers and outcome assessment (Fig. 2). The first study about this topic was published in 2009, with not significant increase or decrease was observed in the following years, with a peak of 10 studies published in 2017.

#### Histological results

Thirty-three studies showed positive histological results following the application of blood-derived products, while 4 reports demonstrated no improvement compared to controls (*n* = 2) or detrimental effect of the experimental products (*n* = 2) (Table 1). Studies performed on models of complete or partial Achilles, digital-flexor or patellar tendon ruptures consistently report enhanced appearance [114, 118, 123] with improved Movin and Bonar scores [36, 40, 122], accelerated healing [37, 65, 73, 76, 102] and minimal cartilage formation in tendon mid-portion [114]. Most studies showed faster collagen fibers maturation, with increased organization and density [47, 72, 76, 93, 102, 114, 117, 124], and a reduction of elastic fibers [37, 60, 72]. Improvement in cell morphology and density [37, 102, 118] as well as reduction of neovascularization [37, 72, 76, 114] were described too. Interestingly, while vascularization is reduced at late phase of tendon healing, it is increased in the early phases [13, 72]. Administration of PRP immediately after injury reduced inflammation and improved cell density, collagen fibers organization and epitenon thickness [24]. Overall, when applied to tendon ruptures, blood-derived products induce an acceleration of the initial phases of tendon healing with reduced inflammation and improved cell proliferation/neovascularization, while these parameters tend to be similar between experimental and control groups at the final follow up [47, 65, 76, 117]. Seven studies concerning collagenase-induced tendinopathy showed improvements in terms of fibers organization [17, 26, 45, 119], fibers dimension and neovascularization [26], overall histological appearance [20, 45, 119], while one study did not identify any effect of PRP treatment compared to platelet-derived growth factor-BB or steroids [100]. Increased cellularity was observed early after treatment in one study [17]. Leukocyte-rich PRP provided better histological results compared to leukocyte-poor formulations in one study [56]. Studies using rotator cuff repair models reported controversial results, ranging from no improvements [33] or detrimental effects on cell density and vascularization [22], to the amelioration of histological appearance [31, 64] and fibers organization [31]. Studies involving injury at the enthesis consistently reported improvements in terms of reduced inflammation and vascularity, [49] deposition of type I and type II collagen [126], fibers organization [115] and histologic appearance [127] following blood-derived products application. Only one study reported a detrimental effect of platelet-rich fibrin matrix on tissue healing with formation of fibrotic tissue [50]. The repair with fibrotic tissue may be the consequence of an excess of stimulation by growth factors resulting in an unnaturally rapid healing response. Thus, while treatment was indeed able to accelerate healing, the dosage need to be fine-tuned to ensure a high-quality tissue repair [57, 116].

**Table 1:** Summary of the effects reported in preclinical studies in different tendon conditions

Condition	Blood-derived products	Bone marrow-derived products	Adipose tissue-derived products
Complete/partial ruptures	Improved histologic appearance and ECM deposition (16/16) Reduction of inflammatory markers (5/5) Improvement of biomechanical properties (8/12)	Improved histologic appearance and ECM deposition (2/3)	Improved histologic appearance and ECM deposition (5/6) Improved biomechanical properties (10/11) Improved MRI appearance (1/3) Improved functional outcomes (2/2)
Tendinopathies	Improved histologic appearance (7/8) Improvement of biomechanical properties (1/2) Improved US and MRI appearance (3/3)	Improved histologic appearance (1/1) Improvement of biomechanical properties (1/1) Reduction of MMPs expression (2/2) Improved functional outcomes (1/1)	Improved histologic appearance and ECM deposition (4/4) Improved biomechanical properties (2/2)
Rotator Cuff Tears	Improved histologic appearance (2/4) Improved biomechanical properties (mid-term follow-up) (7/7) Improvements identified by imaging and functional assessment (1/1)	Improved histologic appearance (1/1) Improvement of biomechanical properties (1/1)	Improved histologic appearance (3/4) Improved biomechanical properties (4/5) Improved MRI appearance (1/1) Improved functional outcome (1/1)
Lesion of the enthesis	Improved histologic appearance and ECM deposition (4/5) Improved biomechanical properties (3/3)	Improved histologic appearance and ECM deposition (1/1) Improved biomechanical properties (4/4) Improved radiographic appearance (1/1) Improved functional outcomes (1/1)	Improved histologic appearance and ECM deposition (4/5) Improvement observed by microCT and Rx (2/2)

*ECM* extracellular matrix, *GFs* growth factors, *mCT* microcomputed tomography, *Rx* X-ray

Number in parentheses reports the fraction of studies confirming the results among total number of studies assessing the specific outcome (studies with positive results/number of studies)

#### Biomechanical results

Enhancement of biomechanical properties of the tissue was obtained in 20 studies, while in 5 cases no improvements were observed compared to controls (Table 1). Controversial results were observed especially in regards of the treatment of tendon ruptures, where 4 studies showed no improvements after treatment [43, 62, 122, 124]. Conversely, maximum failure load [60, 78, 101, 117], tensile strength [47, 93, 118, 123] and mechanical stress [60] improved in treated tendons according to 8 studies. Three studies investigating enthesis repair consistently reported increased strength in the treated tendons [48, 80, 127]. Two studies conducted in collagenase induced tendinopathy models were investigated, one reporting improvements after treatment [45] and the other showing no effect of PRP [100]. Reports about rotator cuff repair models consistently showed improved biomechanical properties [22] in terms of maximum failure load [31, 33, 50, 81, 115], resistance to mechanical stress [50], tensile strength [49] and stiffness [31, 33, 50]. Notably the use of frozen platelet concentrates would not allow to obtain the same results as fresh products [61, 127]. In addition, leukoocyte-rich PRP provides higher improvements in the maximum failure load compared to leukoocyte-poor PRP [56]. Together, these observations suggest that a low rate of live cells, reduced during processing or by freeze/thaw cycles, may correlate with a reduced treatment effectiveness. Interestingly, the contemporary use of NSAIDs and PRP did not provide improvements in terms of maximum failure load compared to PRP-only treated animals [81].

#### Modulation of gene expression, protein production and molecular pathways

The treatment with blood-derived products was able to enhance the gene expression and protein deposition of collagen type I, as reported by 8 different studies involving both tendon rupture models and collagenase-induced tendinopathy [20, 45–47, 56, 60, 74, 119]. This effect, together with increased expression of Scleraxis and Tenascin, appears to be mediated by the FAK/ERK1,2 cascade [20]. Conversely, no consensus was observed concerning collagenase type III expression after treatment with blood derived products [60, 74], even if reduction was frequently reported [45, 46, 119]. Metalloproteases (MMPs) expression appeared reduced after treatment in collagenase-induced tendinopathy [45, 46, 119] and ex vivo experiments [14, 79] but not in tendon ruptures [60]. Reduction of inflammatory mediators such as IL-6 [45, 46, 119], TNFα, IL-8, IL-6 [92] and PGE2 [125] were frequently reported after treatment with blood-derived products. In particular, the inhibition of PGE2 appeared to be mediated by HGF (known component of PRP), leading to a reduced expression of downstream inflammatory effectors such as COX1 and COX2 [125]. The expression of the growth factors TGFβ and IGF, as well as the tendon specific marker TNMD, is increased in the first phases of tendon healing in treated animals, whilst they all decreased at later time-points [47, 60, 73–75]. In a model of rotator cuff tear, an increase in BMP-2 after treatment, possibly benefitting the healing of the bone-tendon interface was reported [115].

#### Imaging and behavior evaluations

Imaging techniques and behavior assessments demonstrated overall positive results using blood-derived products for the treatment of tendon-bone injury, tendon ruptures and collagenase induced tendinopathy, with 7 studies reporting satisfactory results and 1 showing no improvement compared to controls (Table 1). Blood-derived treatments were able to sustain healing, reduce neovascularization, improve tendon structure, and bone formation at the enthesis as observed by ultrasound [26, 114], MRI T2 mapping [40] and micro-CT [127] evaluations. Conflicting results were observed in term of the effect of leukocyte-rich and leukocyte-poor PRP, assessed by MRI T2 mapping, with one study showing better results in tendons treated with leucocyte poor-PRP compared to leucocyte rich-PRP treated samples [119], and another study showing the opposite [56]. Veterinary case series in dogs with spontaneous rotator cuff tendinopathy confirmed the positive results in terms of ultrasound-evaluated echogenicity and heterogeneity correlating with improvements in owner-assessed function score [51]. In a spontaneous model of calcaneal tendon rupture, the use of PRP effectively improved function and quality of life owner-assessed scores, as well as restoring the limb native anatomical condition [96].

### Bone marrow-derived products

#### Selected studies

**Fig. 3:**
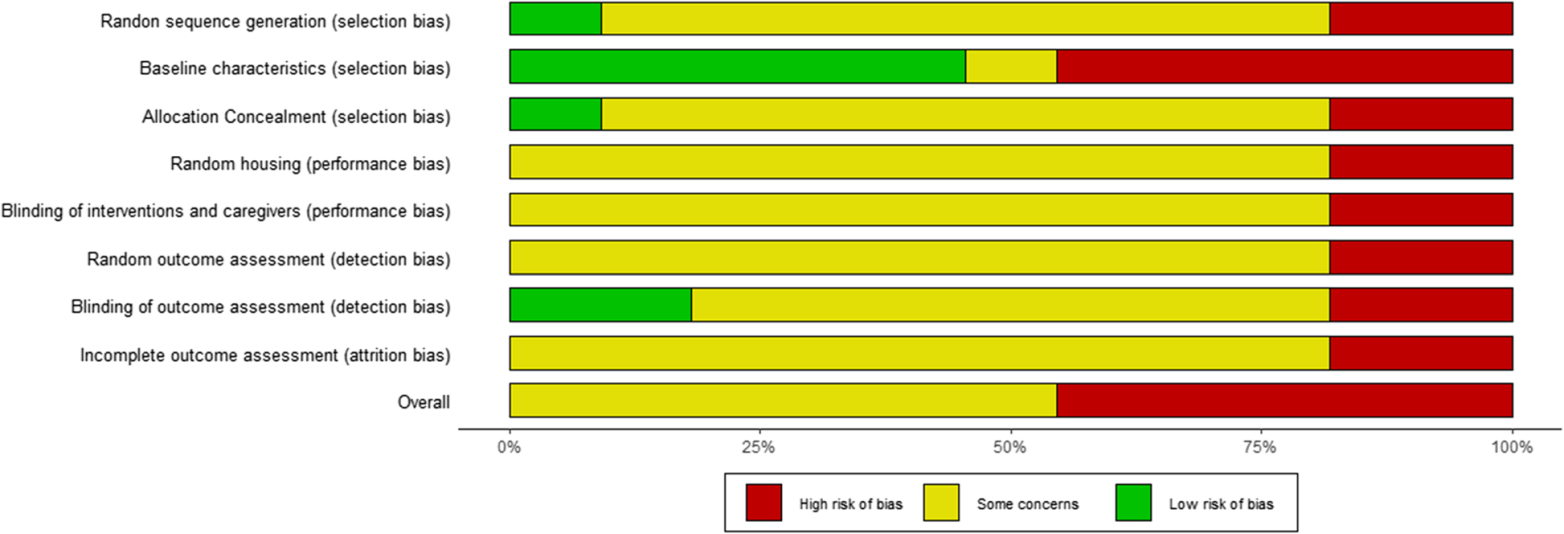
SYRCLE’s risk of bias assessment for in vivo studies investigating the use of bone marrow-derived products for the treatment of tendon pathologies

Rabbit models were the most used across the 11 selected studies (*n* = 4), followed by horse (*n* = 3), rat (*n* = 2) mouse (*n* = 1) and dog models (*n* = 1). Concerning the pathological models, 3 focused on tendon ruptures, 2 on tendinopathy,2 on rotator cuff tears and 3 studies addressed lesions of the enthesis. Six studies employed BMAC, comprising 1 study combining this product with a scaffold and another study combining it with PRP. Five studies used in vitro cultured bone marrow (BM)-MSCs, 4 of which in combination with scaffolds or fibrin glue. The analysis performed using SYRCLE’s Risk of Bias tool, identified 5 studies at high risk of bias, due to the lack of a control group (*n* = 2) or differences between groups at baseline (*n* = 3). Only 1 randomized study and 2 studies with blinded outcome assessors were identified among the selected studies (Fig. 3). The reports about the application of bone marrow-based product in tendon pathologies are generally older than articles about blood-derived products, with the first study published in 2002, and only 5 studies published after 2012.

#### Histology and immunohistochemistry

Overall, 7 studies reported positive results in histological appearance of tendons treated with bone marrow-derived products, while 1 study reported a detrimental effect. One study showed mixed results (Table 1). In the context of tendon ruptures, treatments based on scaffolds seeded with cultured bone marrow-MSCs demonstrated superior cell proliferation and improved tissue formation compared to controls, especially in the initial phases of tendon healing [32, 87], with improved deposition of collagen type I and type III. Nevertheless, at the final stage the repaired tissue appeared to be similar to controls [87]. Other studies reported the ectopic formation of bone in some samples, rising questions about the appropriateness of the procedure [7]. Studies investigating tendon-to-bone repair using BM-MSCs with scaffolds or BMAC added with bone morphogenetic protein-2 (BMP-2) showed improved integration, mineralization [54], bone appearance and density [63, 104], increased fibrocartilage formation [104], deposition of collagen type II and presence of SOX9^+^ cells [129] compared to controls. One study reported an increased presence of macrophages in treated tendons [129], suggesting possible enhancement of immune reaction. BMAC applied to rotator cuff disorders improved fibers continuity and orientation [69]. In a horse spontaneous model of digital flexor tendon disorder, the use of BM-MSCs in marrow supernatant was able to improve the histological appearance in terms of vascularity, cell density, fiber organization, as well as GAG and water content [99] compared to saline.

#### Biomechanical results

Positive biomechanical results were reported by all the six studies that included this kind of evaluation following administration of bone marrow-derived products for tendon pathologies (Table 1). The effects on the biomechanical properties of tendons appeared to be related to an improvement of maximum stress/load to failure and tensile stiffness in different contexts including experimental defects of the tendon mid-portion and of tendon-bone junction [7, 54, 63, 87]. These results were obtained with either cultured cells seeded on scaffolds or BMAC. BMAC was able to improve ultimate load to failure of teared rotator cuff in a rabbit model [69], as well as structural stiffness in a naturally occurring injury of digital flexor tendon in horses [99]. Elasticity was not affected by treatment with BM-derived products [32, 99].

#### Molecular pathways

BMAC was able to induce the expression of collagen type I, decorin and COMP in horse digital flexor tendon at comparable levels with respect to blood derived products (PRP, PPP, serum), while contemporary reducing the expression of collagen type III, MMP-3, MMP-13 [95, 99]. The observed differences between BMAC and PRP could be explained by the higher content of growth factors in BMAC, compared to blood-derived products [69].

#### Imaging and functional outcomes

All studies evaluating imaging (*n* = 1) or behavior/functional (*n* = 2) outcomes of BM-derived products for tendon pathologies reported positive results (Table 1). Scaffolds seeded with BM-MSCs were are able to improve the radiographic appearance and bone mineral density in a model of enthesis injury [104]. Dogs receiving autologous cancellous bone scaffold supplemented with bone marrow for the reconstruction of tendon-to-bone junction showed a 90% recovery of preoperative weight bearing at 16 weeks after surgery [54]. Treatment with freshly isolated BM-MSCs added to PRP was able to improve lameness and allowed for returning to activity in 85% of race horses affected by spontaneous suspensory ligament desmopathy or superficial flexor [105].

### Adipose tissue-derived products

#### Selected studies

**Fig. 4:**
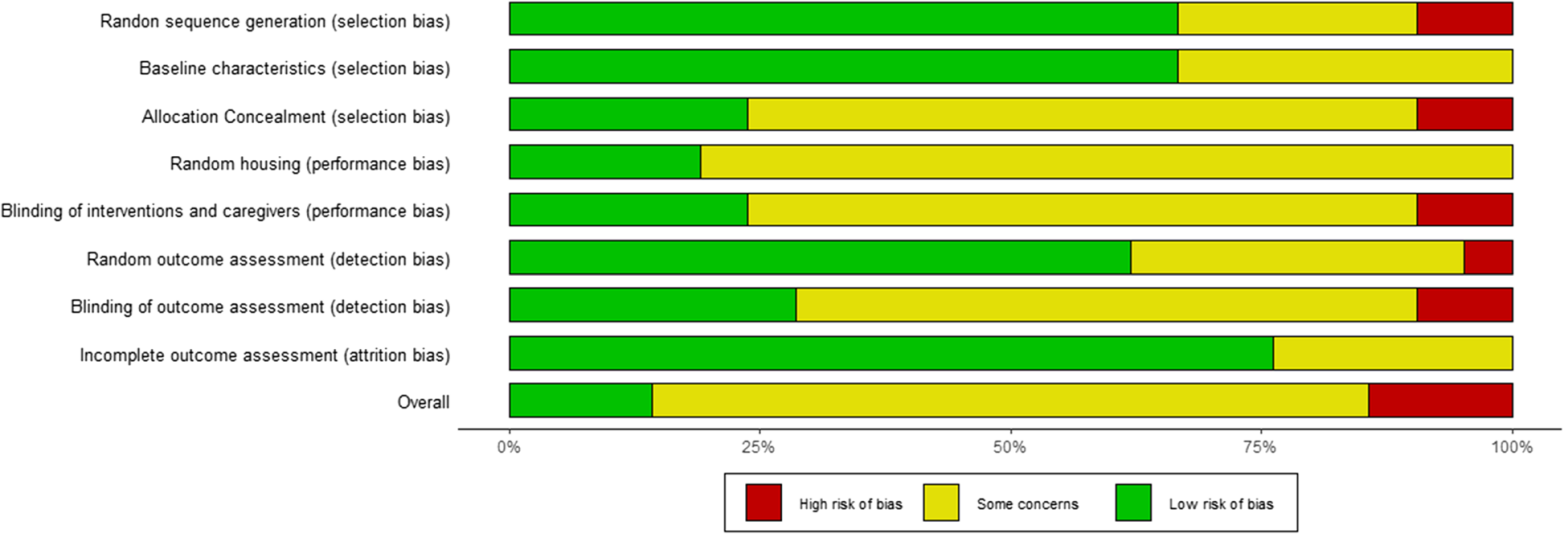
SYRCLE’s risk of bias assessment for in vivo studies investigating the use of adipose tissue-derived products for the treatment of tendon pathologies

Most of the studies concerning adipose-derived products were conducted in rats, followed by rabbits (*n* = 4), horses (*n* = 2), mice (*n* = 1) and sheep (*n* = 1). Eleven studies addressed models of tendon ruptures, 5 focused on spontaneous or experimentally-induced tendinopathy and 5 focused on lesions of the tendon-bone junctions. In 5 cases, the site of injury was the supraspinatus tendon (2 tendon ruptures, one collagenase induced tendinopathy, one detachment of tendon from bone). Cultured adipose derived mesenchymal stem cells (ASCs) were frequently used either alone (*n* = 8) or in combination with scaffolds/hydrogels (*n* = 6) or with other treatments (biophysical stimulation, Vitamin D, PDGF; *n* = 3). Four studies used freshly isolated stromal vascular fraction (SVF) alone (*n* = 2) or in combination with fibrin glue (*n* = 2). Risk of bias analysis performed using SYRCLE’s tool demonstrated high risk only in 3 studies, due to lack of randomization and blinding of outcome assessment. Notably the frequency of randomized studies is higher among reports investigating adipose-derived products compared to studies about blood- and bone marrow-derived products (Fig. 4). The time-distribution of these studies resembles the one observed for blood-derived products, with the first article published in 2011 and no clear trend afterwards.

#### Histology and immunohistochemistry

Positive histological findings after treatment of diseased tendons with adipose tissue-derived products were found by 15 studies, while in 2 cases no improvements compared to controls were reported (Table 1). Studies reporting the use of ASCs seeded on scaffolds or associated to surgical repair for the treatment of tendon ruptures showed improved morphology [66, 120], fibers organization and collagen deposition [11, 29], or reduced fatty infiltration [85]. ASCs were able to effectively colonize decellularized scaffolds, and this is considered crucial to allow scaffold integration and healing [8]. Only one study used ASCs without scaffold or surgery for tendon repair, reporting no differences between treated and control samples [42]. Four studies applying ASCs treatment in experimental models of collagenase-induced tendinopathy demonstrated reduced inflammation [19] and cellularity [35], improvement of tissue appearance, Bonar score, fibers thickness and organization [19, 35, 59, 86], with reduced neovascularization and tissue degeneration [35, 86]. Five studies investigated the role of SVF or ASCs in the treatment of tendon-bone junction injuries, using surgical repair and scaffolds. Four of them showed improved formation of mature fibrocartilage with enhanced collagen deposition [18, 80, 97], increase in the Collagen type I/type III ratio and improved tissue appearance [71]. One study reported a reduction of inflammation with no effect on matrix organization [108]. Noteworthy, these surgical studies showed that ASCs are able to colonize scaffolds and to differentiate into osteoblast/chondrocytes, providing a direct contribution to tissue healing [18, 80].

#### Biomechanical tests

Fifteen studies showed improvements in biomechanical properties of injured tendons treated with adipose tissue-derived products, while 2 studies failed to detect improvements compared to controls (Table 1). The use of ASCs-based treatments allowed for the improvement of the biochemical properties of injured tendons in several pre-clinical models of tendon ruptures and tendon-bone detachment [66, 97, 120]. ASCs and SVF in combination with surgical repair and/or scaffolds improved maximum load [10, 11, 18, 29, 71, 80, 85], stiffness [10, 18, 29, 70, 71], tensile strength [70, 107], energy absorption [10, 11, 108] and mechanical deformation capacity of treated tendons [108]. Two studies reported no differences in mechanical properties after treatments, one using ASCs-seeded scaffolds [8] and one using ASCs alone [42]. Two studies concerning models of collagenase induced-tendinopathy showed improvements in maximum load and stiffness after ASCs injections [19, 21].

#### Molecular pathways

ASCs and SVF were able to enhance the deposition of collagen type I and collagen type I/type III ratio, alone or in association to scaffolds and surgical repair [21, 66, 71, 86, 107]. One study reported an increase in collagen cross-linking with hydroxylysylpyridinoline after treatment with ASCs [42], while others showed improvements in the transcription of the tendon specific genes scleraxis and tenascin [21, 66], as well as of FGF and VEGF [107]. Reduction of the pro-fibrotic factor TGFβ was reported in a model of Achilles tendon lesion treated with ASCs and surgical repair [107]. Increased BMP-2 expression was observed after treatment with ASCs in a model of injured enthesis, possibly improving healing of tendon-bone junction injuries [71].

#### Imaging and behavior

Improvements in functional outcomes after treatment with adipose tissue-derived products were described by 2 studies. Five studies assessed treated tendons with imaging techniques, each reporting various results, ranging from positive to detrimental effect (Table 1). SVF was able to improve MRI signal-to-noise at 12 weeks after surgical repair in a model of supraspinatus and patellar tendon lesions, especially at longer follow up times [70]. Two other studies using ASCs and SVF reported controversial results, with either no differences in terms of ultrasound parameters [42], or even increased inflammation and lesion size in treated tendons [120]. One study showed that ASCs remained at the treatment site at medium terms after injection, suggesting possible prolonged action [41]. A limited number studies identified functional improvements in terms of Achilles functional index (AFI), pow print intensity, stance time and duty cycle in experimental rats treated with ASCs [8, 120]. ASCs treatment demonstrated efficacy in enhancing bone volume, trabecular number and thickness by micro-CT [18, 97], and in improving bone formation observed by radiographic evaluation [18] in models of tendon-bone junction injury.

## Discussion

Overall, the findings of the studies investigating the effects of blood-, bone marrow- and adipose tissue-derived products for the treatment of tendon pathologies demonstrated that they are able to support tissue healing in a variety of experimental and clinical settings, with measurable improvements in histological, biomechanical, molecular, functional and imaging outcomes.

Regardless the specific condition, both surgical and conservative therapies for tendon injuries are characterized by frequent failure and relapses, as well as long recovery time [68, 77, 94]. Then, the rationale behind the use of orthobiologics relies on the possibility to accelerate tissue healing while at the same time improving the quality. In fact, several aspects of orthobiologics action reported by the studies analyzed appear to address specific mechanisms in tendon healing that span across 3 continuous phases: inflammation, proliferation and remodeling [30, 111]. In the first stage, immune cells are recruited to the injury site, with activation of platelets and tissue resident progenitors. Tissue progenitors may directly differentiate into mature tenocytes to substitute cellular loss or they aid healing by orchestrating the repair process through the production of soluble mediators [53]. This phase involves different cytokines and growth factors, including IL-1β, IL-6, bFGF, IGF1, TGFβ, VEGF and PDGF [30], that are highly concentrated in blood-derived products, especially VEGF. A timely regulation of neovascularization is of crucial importance in tendon healing since it allows for rapid cell and platelet recruitment, while preventing the formation of necrotic tissue immediately after injury [82, 103]. Inflammation should then progressively decrease, and this is possibly aided by the PRP action, which inhibits the production of pro-inflammatory cytokines (IL-6, TNFα, PGE2) in later stages. The control of inflammation is considered a key features of mesenchymal stem cells from both bone marrow and adipose tissue [84], but this effect is under-reported in the studies analyzed. During the proliferation phase of tendon healing, mitosis of tissue progenitors and migration of recruited cells cause an increase in cell density; ECM deposition is initiated in a non-organized manner, with prevalence of collagen type III, proteoglycans and fibronectin [30, 109]. Tendons treated with orthobiologics demonstrated enhanced cell content and proliferation with respect to controls, and the contribution of these products in terms of growth factors - especially PDGF, IGF and FGF - represent the putative mechanism of this effect [30]. At the same time, growth factors contained within by orthobiologics would explain the increase in terms of ECM elements (collagen type I, II, III and GAG) observed in treated tendons compared to controls [90, 128]. Although an excess of growth factors bears the risk of forming fibrotic tissue, to some extent this is expected to occur during the proliferative phase [38, 109]. Indeed, the tissue and ECM remodeling phase, which is crucial for the maturation of repaired tissue and the restoration of proper tendon, should follow this stage. In this phase, collagen type III is replaced by collagen type I and the collagen fibers form cross-links [109], both events that are stimulated by orthobiologics. In addition, adipose-derived products showed the ability to increase the expression of tendon specific markers such as SCX and tenascin, favoring tissue maturation by stimulating progenitor cell differentiation into tenocytes [15, 21, 66]. Indeed, the action on tissue resident progenitors is a well-known mechanism of action of the orthobiologics, supporting the use of regenerative medicine as treatment for degenerative pathologies [89, 110]. Metalloproteases play a relevant role in the ECM-remodeling phase of physiological tendon healing. They are finely regulated in physiological conditions, but in presence of chronic inflammation a dysregulation of their expression may foster ECM degeneration and pathology progression [27]. Indeed, especially in the context of tendinopathy their inhibition allows for counteracting pathology progression and promote tissue healing [83].

The effects of each product in the treatment of tendon pathologies are summarized in Table 1. Considering the different tendon condition, different applications of orthobiologics should be considered. In case of tendon complete ruptures the use of orthobiologics in combination with surgical repair and/or scaffolds appear superior to that of orthobiologics alone. Otherwise, the use of these products for the treatment of partial ruptures showed satisfactory results even without scaffold or surgical repair. Improvements of the histologic appearance and enhancement of ECM deposition (especially collagen type I) were reported for all products, while cell-based approaches, but not blood-derived products, allowed to observe improvements in the biomechanical properties of repaired tissue. Interestingly, the lack of improvements in biomechanical properties after treatment with blood-derived products was confirmed across a variety of models, techniques and injury sites. Adipose-derived products were reported to improve imaging and functional outcomes. Ectopic bone formation was identified as a possible side effect of orthobiologics, especially in bone marrow-derived products, while adipose-based treatments were reported to reduce fibrosis. In general, the application of blood-derived products seems to accelerate healing, demonstrating superior outcomes at short follow up and similar results compared to control in the long term. On the contrary, cell-based treatments were reported to produce more durable results in terms of quality of the repaired tissue.

In the context of experimentally-induced or spontaneous tendinopathy, histological improvements were reported for all orthobiologics, while functional improvements were reported only after cell-based treatments. In particular the use of adipose-derived products enhanced the biomechanical properties of the repaired tissue as well as the deposition of tendon ECM.

Concerning rotator cuff disease, blood-derived products were reported to effectively improve imaging appearance and functional scores in treated animals. This treatment also ameliorated the biochemical properties of tendons at short-term, but not at longer follow-ups. This observation supports the hypothesis that PRP and similar products effectively accelerate healing rather than improving the final outcome. Cell-based treatment were able to improve both histological appearance and biomechanical properties of repaired tendons.

Orthobiologics appear to be especially effective in the treatment of enthesis injuries. Indeed, all products were able to improve histologic appearance, ECM deposition and biomechanical properties of repaired tendons. This is possibly due to the positive effects on fibrocartilage and bone tissue formation. In fact, while the ectopic formation of fibrocartilage or bone would represent a drawback for orthobiologics application to tendons mid-portion injuries, the increased production of elements such as collagen type II, proteoglycans and BMP-2, would have beneficial effects in the context of the enthesis.

Several formulations of each product are available, especially concerning blood-based products. Interestingly, leukocyte rich PRP was reported to induce better results compared to leucocyte-poor counterparts. In addition, frozen/thawed PRP has limited effectiveness compared to the fresh product. Interestingly, the additional use of NSAIDs did not modify PRP effects.

Among the limitations of the present work, data about cell-based therapies are reported in a low number of studies, frequently focusing on cultured mesenchymal stem cells rather than products obtained at point of care. Thus, the effectiveness demonstrated by these approaches has limited generalizability. On the contrary, the evidence for blood-derived products is based on a large pool of studies, with consistent results. Another limitation is due to the lack of consensus about pathological models and type of product administration throughout literature, preventing the elaboration of defined strategies for the use of orthobiologics in a preclinical setting. Furthermore, the low quality of research reports, especially concerning bone marrow-derived products, may have biased the results of this systematic review.

## Conclusion

Overall, the preclinical results about orthobiologics applications to tendon pathologies support the rationale of their clinical use, and encourage the performance of clinical trials aimed to confirm these results in human subjects. The effect of blood-derived products appears to be related to an acceleration of the healing process, without improvements in the final structure and properties of repaired tendon. Cell-based products have been reported to produce more durable results, although with a lower level of supporting evidences. 

In particular, many reports about cell-therapies focused on the use of cultured MSCs rather than products obtained at the point of care. In consideration of the regulatory, safety-related and economic limitations to the use of cultured cells in the clinical practice, future pre-clinical trials should focus on minimally manipulated products (i.e. BMAC or SVF). Possible safety issues about the use of orthobiologics for tendon pathologies are related to the formation of fibrotic tissue (fibrocartilage) and ectopic bone. On the other hand, this possible side effect turns into an advantage when treating tendon-bone junction injuries, as confirmed by studies assessing enthesis repair.

## Supplementary Information

**Additional file 1.**

## Data Availability

N/A.
