# Supplementary material for: The effects of orthobiologics in the treatment of tendon pathologies: a systematic review of preclinical evidence
Source: J Exp Orthop. 2022 Apr 8;9:31. doi: 10.1186/s40634-022-00468-w (PMC8994001; doi:10.1186/s40634-022-00468-w)
Supplement: Supplementary file 1 — Additional file 1. [file 40634_2022_468_MOESM1_ESM.docx]

Supplementary Table I. Keywords

| **Platelet-rich plasma** | |
| --- | --- |
| MEDLINE | (("platelet rich plasma"[MeSH Terms]) AND ((((((((tendinopathies[MeSH Terms]) OR (tendinopathy[MeSH Terms])) OR (tendinitis[MeSH Terms])) OR (tendinitides[MeSH Terms])) OR (patellar tendon[MeSH Terms])) OR (achilles tendon[MeSH Terms])) OR (achilles tendons[MeSH Terms])) OR (cuff, rotator[MeSH Terms]))) AND (((((((animal model) OR (rat)) OR (rabbit)) OR (dog)) OR (sheep)) OR (mouse)) OR (in vivo)) |
| Web of Science | #1 ((((((ALL=(animal model)) OR ALL=(rat)) OR ALL=(rabbit)) OR ALL=(sheep)) OR ALL=(dog)) OR ALL=(horse)) OR ALL=(in vivo)  #2 ((((((ALL=(tendon)) OR ALL=(tendinitis)) OR ALL=(tendinopathy)) OR ALL=(achilles)) OR ALL=(patellar)) OR ALL=(elbow)) OR ALL=(rotator cuff)  #3 ALL=(platelet rich plasma)  ((#1) AND #2) AND #3 |
| EMBASE | ('platelet rich plasma'/exp OR 'platelet rich plasma') AND ('tendon'/exp OR tendon OR 'tendinitis'/exp OR tendinitis) AND ('in vivo study'/exp OR 'in vivo study' OR 'animal model'/exp OR 'animal model' OR 'rat'/exp OR rat OR 'dog'/exp OR dog OR 'sheep'/exp OR sheep OR 'horse'/exp OR horse OR 'rabbit'/exp OR rabbit) |
| **BMAC/MFX/BMSCs** | |
| MEDLINE | ((((((((animal model) OR (rat)) OR (rabbit)) OR (dog)) OR (sheep)) OR (mouse)) OR (in vivo)) AND ((((((((tendinopathies[MeSH Terms]) OR (tendinopathy[MeSH Terms])) OR (tendinitis[MeSH Terms])) OR (tendinitides[MeSH Terms])) OR (patellar tendon[MeSH Terms])) OR (achilles tendon[MeSH Terms])) OR (achilles tendons[MeSH Terms])) OR (cuff, rotator[MeSH Terms]))) AND (((((bone marrow[MeSH Terms]) OR (BMAC)) OR (bone marrow concentrate)) OR (bone marrow aspirate)) OR (BMA)) |
| Web of Science | #1 ((((((ALL=(animal model)) OR ALL=(rat)) OR ALL=(rabbit)) OR ALL=(sheep)) OR ALL=(dog)) OR ALL=(horse)) OR ALL=(in vivo)  #2 ((((((ALL=(tendon)) OR ALL=(tendinitis)) OR ALL=(tendinopathy)) OR ALL=(achilles)) OR ALL=(patellar)) OR ALL=(elbow)) OR ALL=(rotator cuff)  #3 ((((ALL=(bone marrow)) OR ALL=(bone marrow concentrate)) OR ALL=(bone marrow aspirate)) OR ALL=(BMAC)) OR ALL=(BMA)  ((#1) AND #2) AND #3 |
| EMBASE | ('bone marrow'/exp OR 'bone marrow' OR 'bone marrow concentrate'/exp OR 'bone marrow concentrate' OR 'bone marrow aspirate'/exp OR 'bone marrow aspirate') AND ('tendon'/exp OR tendon OR 'tendinitis'/exp OR tendinitis) AND ('in vivo study'/exp OR 'in vivo study' OR 'animal model'/exp OR 'animal model' OR 'rat'/exp OR rat OR 'dog'/exp OR dog OR 'sheep'/exp OR sheep OR 'horse'/exp OR horse OR 'rabbit'/exp OR rabbit) |
| **SVF/mFAT/ASCs** | |
| MEDLINE | ((((((((animal model) OR (rat)) OR (rabbit)) OR (dog)) OR (sheep)) OR (mouse)) OR (in vivo)) AND ((((((((tendinopathies[MeSH Terms]) OR (tendinopathy[MeSH Terms])) OR (tendinitis[MeSH Terms])) OR (tendinitides[MeSH Terms])) OR (patellar tendon[MeSH Terms])) OR (achilles tendon[MeSH Terms])) OR (achilles tendons[MeSH Terms])) OR (cuff, rotator[MeSH Terms]))) AND (((((SVF) OR (stromal vascular fraction)) OR (micro-fragmented)) OR (microfragmented)) OR (adipose tissue)) |
| Web of Science | #1 ((((((ALL=(animal model)) OR ALL=(rat)) OR ALL=(rabbit)) OR ALL=(sheep)) OR ALL=(dog)) OR ALL=(horse)) OR ALL=(in vivo)  #2 ((((((ALL=(tendon)) OR ALL=(tendinitis)) OR ALL=(tendinopathy)) OR ALL=(achilles)) OR ALL=(patellar)) OR ALL=(elbow)) OR ALL=(rotator cuff)  #3 (((((ALL=(stromal vascular fraction)) OR ALL=(microfragmented )) OR ALL=(adipose tissue)) OR ALL=(micro-fragmented))  ((#1) AND #2) AND #3 |
| EMBASE | ('stromal vascular fraction'/exp OR 'stromal vascular fraction' OR 'adipose tissue'/exp OR 'adipose tissue' OR 'microfragmented adipose tissue'/exp OR 'microfragmented adipose tissue') AND ('tendon'/exp OR tendon OR 'tendinitis'/exp OR tendinitis) AND ('in vivo study'/exp OR 'in vivo study' OR 'animal model'/exp OR 'animal model' OR 'rat'/exp OR rat OR 'dog'/exp OR dog OR 'sheep'/exp OR sheep OR 'horse'/exp OR horse OR 'rabbit'/exp OR rabbit) |
